# Prediction of protein submitochondria locations by hybridizing pseudo-amino acid composition with various physicochemical features of segmented sequence

**DOI:** 10.1186/1471-2105-7-518

**Published:** 2006-11-30

**Authors:** Pufeng Du, Yanda Li

**Affiliations:** 1 Bioinformatics Division, TNLIST and Department of Automation, Tsinghua University, Beijing, 100084, China

## Abstract

**Background:**

Knowing the submitochondria localization of a mitochondria protein is an important step to understand its function. We develop a method which is based on an extended version of pseudo-amino acid composition to predict the protein localization within mitochondria. This work goes one step further than predicting protein subcellular location. We also try to predict the membrane protein type for mitochondrial inner membrane proteins.

**Results:**

By using leave-one-out cross validation, the prediction accuracy is 85.5% for inner membrane, 94.5% for matrix and 51.2% for outer membrane. The overall prediction accuracy for submitochondria location prediction is 85.2%. For proteins predicted to localize at inner membrane, the accuracy is 94.6% for membrane protein type prediction.

**Conclusion:**

Our method is an effective method for predicting protein submitochondria location. But even with our method or the methods at subcellular level, the prediction of protein submitochondria location is still a challenging problem. The online service SubMito is now available at:

## Background

Mitochondria are subcellular organelles that appear only in eukaryotic cells. They are surrounded by two layers of membrane, the inner membrane and the outer membrane. Proteins which are localized within mitochondria play important roles in energy metabolism process. Inner membrane, outer membrane and matrix contain proteins which contribute to different procedures in energy metabolism. It has been proved that mitochondria are involved in several complex biological processes, like programmed cell death[1] and ionic homeostasis[2]. There are over 100 kinds of complex diseases related with mitochondria. Thus, it is important to understand the protein function within mitochondria.

Knowing protein localization is an important step to understand its function. But, to experimentally identify the protein subcellular location is costly and time consuming. A host of computational systems which are designed for predicting protein subcellular location had been developed during the last two decades. Various features of sequence had been used for predicting protein subcellular location, such as terminal signalling peptides[3,4], amino acid composition [5-8], pseudo-amino acid composition[9,10], dipeptide composition[11,12], functional domain composition[13,14] and GO information[14,15]. And a number of machine learning approaches had been introduced to predict protein subcellular location, such as the Markov chain method[16], discriminate function[17,18], SVM[9,19-21], artificial neural network[22,23], OET-KNN[24], fuzzy-KNN[11] and classifier fusion technique [24-26]. Some reviews described most of these methods in detail[27,28]. Most of these methods assigned a unique subcellular location for a protein. But other methods can assign more than one subcellular locations for a protein [29-31], which are called multiplex subcellular location predictors.

Recently, the advances of experimental technology have enabled the large-scale identification of nuclear proteins[32,33]. A database for nuclear proteins and their subnuclear location has been constructed[34]. The prediction of protein subcellular location has been extended to a new level, the subnuclear level[35,36], where the protein location within cell nucleus can be predicted.

To the best of our knowledge, however, there exists no computational system for predicting protein submitochondria location. In this paper, we develop a computational system called SubMito to predict the submitochondria location for a protein only from its primary sequence. The system can assign one of the three submitochondria locations which are mitochondria inner membrane, mitochondria outer membrane and mitochondria matrix for a sequence. Since there had been several sophisticated methods for predicting mitochondria protein, like MitoPred[37], this prediction that goes one level deeper should be a good complement to the mitochondrial protein identification systems.

Membrane protein type prediction is another challenging problem. Some powerful methods [38-45] have been introduced to predict membrane protein type for a membrane protein. We try to integrate membrane protein type prediction with submitochondria location prediction. We predict the membrane protein type for a protein after we predict it to be a membrane protein. Due to the limitation of the data, we only predict membrane protein type for mitochondrial inner membrane proteins.

We hope that our work can provide a useful complement to those subcellular location predictors which are developed previously.

## Results

### Evaluation method

Since the leave-one-out cross validation method is more objective and rigorous[27] than sub-sampling methods, we adopt leave-one-out cross validation method in our work to get a more accurate estimation of prediction accuracy and Matthew's correlation coefficient[46] which are widely used statistics for evaluating the performance of subcellular location predictors.

The prediction accuracy and Matthew's correlation coefficient of the ith location are defined in equation 1 and equation 2 respectively.

ACC(i)=TP(i)TP(i)+FN(i)     (1)
 MathType@MTEF@5@5@+=feaafiart1ev1aaatCvAUfKttLearuWrP9MDH5MBPbIqV92AaeXatLxBI9gBaebbnrfifHhDYfgasaacH8akY=wiFfYdH8Gipec8Eeeu0xXdbba9frFj0=OqFfea0dXdd9vqai=hGuQ8kuc9pgc9s8qqaq=dirpe0xb9q8qiLsFr0=vr0=vr0dc8meaabaqaciaacaGaaeqabaqabeGadaaakeaacqWGbbqqcqWGdbWqcqWGdbWqcqGGOaakcqWGPbqAcqGGPaqkcqGH9aqpdaWcaaqaaiabdsfaujabdcfaqjabcIcaOiabdMgaPjabcMcaPaqaaiabdsfaujabdcfaqjabcIcaOiabdMgaPjabcMcaPiabgUcaRiabdAeagjabd6eaojabcIcaOiabdMgaPjabcMcaPaaacaWLjaGaaCzcamaabmaabaGaeGymaedacaGLOaGaayzkaaaaaa@48AC@

MCC(i)=TP(i)TN(i)−FP(i)FN(i)(TP(i)+FP(i))(TP(i)+FN(i))(TN(i)+FN(i))(TN(i)+FP(i))     (2)
 MathType@MTEF@5@5@+=feaafiart1ev1aaatCvAUfKttLearuWrP9MDH5MBPbIqV92AaeXatLxBI9gBaebbnrfifHhDYfgasaacH8akY=wiFfYdH8Gipec8Eeeu0xXdbba9frFj0=OqFfea0dXdd9vqai=hGuQ8kuc9pgc9s8qqaq=dirpe0xb9q8qiLsFr0=vr0=vr0dc8meaabaqaciaacaGaaeqabaqabeGadaaakeaacqWGnbqtcqWGdbWqcqWGdbWqcqGGOaakcqWGPbqAcqGGPaqkcqGH9aqpdaWcaaqaaiabdsfaujabdcfaqjabcIcaOiabdMgaPjabcMcaPiabdsfaujabd6eaojabcIcaOiabdMgaPjabcMcaPiabgkHiTiabdAeagjabdcfaqjabcIcaOiabdMgaPjabcMcaPiabdAeagjabd6eaojabcIcaOiabdMgaPjabcMcaPaqaamaakaaabaGaeiikaGIaemivaqLaemiuaaLaeiikaGIaemyAaKMaeiykaKIaey4kaSIaemOrayKaemiuaaLaeiikaGIaemyAaKMaeiykaKIaeiykaKIaeiikaGIaemivaqLaemiuaaLaeiikaGIaemyAaKMaeiykaKIaey4kaSIaemOrayKaemOta4KaeiikaGIaemyAaKMaeiykaKIaeiykaKIaeiikaGIaemivaqLaemOta4KaeiikaGIaemyAaKMaeiykaKIaey4kaSIaemOrayKaemOta4KaeiikaGIaemyAaKMaeiykaKIaeiykaKIaeiikaGIaemivaqLaemOta4KaeiikaGIaemyAaKMaeiykaKIaey4kaSIaemOrayKaemiuaaLaeiikaGIaemyAaKMaeiykaKIaeiykaKcaleqaaaaakiaaxMaacaWLjaWaaeWaaeaacqaIYaGmaiaawIcacaGLPaaaaaa@8345@

The overall prediction accuracy is defined in equation 3.

ACCoverall=1N∑k=13TP(k)     (3)
 MathType@MTEF@5@5@+=feaafiart1ev1aaatCvAUfKttLearuWrP9MDH5MBPbIqV92AaeXatLxBI9gBaebbnrfifHhDYfgasaacH8akY=wiFfYdH8Gipec8Eeeu0xXdbba9frFj0=OqFfea0dXdd9vqai=hGuQ8kuc9pgc9s8qqaq=dirpe0xb9q8qiLsFr0=vr0=vr0dc8meaabaqaciaacaGaaeqabaqabeGadaaakeaacqWGbbqqcqWGdbWqcqWGdbWqdaWgaaWcbaGaem4Ba8MaemODayNaemyzauMaemOCaiNaemyyaeMaemiBaWMaemiBaWgabeaakiabg2da9maalaaabaGaeGymaedabaGaemOta4eaamaaqahabaGaemivaqLaemiuaaLaeiikaGIaem4AaSMaeiykaKcaleaacqWGRbWAcqGH9aqpcqaIXaqmaeaacqaIZaWma0GaeyyeIuoakiaaxMaacaWLjaWaaeWaaeaacqaIZaWmaiaawIcacaGLPaaaaaa@4CA0@

*TP*(*i*), *TN*(*i*), *FP*(*i*), *FN*(*i*) are the numbers of true positives, true negatives, false positives and false negatives of the ith location. N is the total number of the sequences in training data set.

### Prediction performance

The leave-one-out cross validation result is shown in Table 1.

**Table 1:** The leave one out cross validation result

Label	Compartment	TP	TN	FP	FN	ACC	MCC
1	Inner membrane	112	173	13	19	85.5%	0.791
2	Outer membrane	21	273	3	20	51.2%	0.636
3	Matrix	137	143	29	8	94.5%	0.774

Overall accuracy		85.2%					

After a sequence is predicted to localize at inner membrane, we continue to predict its membrane protein type. In the correctly identified 112 inner membrane proteins, there are 106 of them predicted to be correct membrane protein type. There are only 6 of them predicted to be wrong membrane protein type. The method correctly predicts the membrane protein type and the submitochondria location for 80.9% of the 131 inner membrane proteins. For different membrane protein types, 84 out of 101 multi-pass inner membrane proteins are predicted correctly, the success rate is about 83.2%; 22 out of the 30 matrix-side membrane protein are predicted correctly, making the success rate about 73.3%.

### Prediction on complete proteome

We adopt our method on the complete sequenced mitochondrial proteome of Arabidopsis thaliana to demonstrate that our method can predict a fraction of protein to different submitochondria locations. The mitochondrial proteome of Arabidopsis thaliana is downloaded from AMPDB[47].

The prediction result is shown in Table 2. Our method predicts that about 21% of all proteins are in the inner membrane, 13% of all proteins are in the outer membrane and 66% of all proteins are in the matrix. The distribution of the prediction result shows that the majority of the Arabidopsis thaliana mitochondria proteome are located at matrix. The experimental research on yeast mitochondria proteome[48] also shows that the majority of the mitochondria proteome are soluble proteins. This observation consists with our prediction results. Because only a very small part of these proteins are annotated with submitochondria location (18 of inner membrane, 11 of outer membrane and 15 of matrix), we can not provide a good estimation for the prediction accuracy on this particular mitochondria proteome. However, our method can correctly identify most of these annotated sequences (10 out of 18 of inner membrane, 11 out of 11 of outer membrane and 15 out of 15 of matrix). This implies that our method should be a novel tool for computational annotating those sequences without submitochondria location annotation.

**Table 2:** Prediction result on complete mitochondria proteome of Arabidopsis thaliana

Locations	Number of sequence	Proportion
Inner membrane	109	21%
Outer membrane	64	13%
Matrix	323	66%

Over all	496	100%

## Discussion

### Result

Because there exists no other method for predicting protein submitochondria location, we are unable to provide a comparison with other methods. We are focusing on different dataset even for the membrane protein type prediction part, so the comparison with other methods on the same basis is impossible. By reviewing the performance that most subcellular location predictors can achieve, we can say that our method has high overall prediction accuracy.

Our method can identify proteins localized at the inner membrane and matrix very well, but identifying the outer membrane proteins does not work as well as the other two locations. For membrane protein type prediction part, our method can correctly predict membrane protein type for 94.6% of the correctly predicted inner membrane protein. The accuracy of the whole cascade prediction is more than 80%. Thus, our method is an effective method for predicting protein submitochondria location and the membrane protein type for mitochondria inner membrane proteins.

We show MCC value in each location in order to show a more comprehensive evaluation of the performance of our predictor. Since MCC considers not only the number of true positives but also the number of false positives, false negatives and true negatives, it is more reliable and more comprehensive than accuracy statistic, especially when the training set is unbalanced. Showing MCC and accuracy together can give the readers a clearer understanding on the performance of our method. The MCC range of 0.6 to 0.7 shows that our method has good prediction performance. And the accuracy we report should not be a result of the problem caused by unbalanced training set.

### Method

As we described in Method section, we set the sequence identity cut off to 40%. As suggested by some recent research[25], the sequence identity should be controlled at level 25% to get rid of the homologues and redundancy bias. But if we use such low cut off value, we can not obtain enough sequences to build sufficient large training set. Thus we use a higher sequence identity cut off value in order to get a balance between the homologues bias and the training set size.

We have tried different segmentation numbers which is the parameter c in our method. The prediction results of c = 1, 2, 3, 4 are shown in Table 3. It is very interesting that the prediction accuracy of every submitochondria location seems to peak at a special c value. Two of these peaks and the overall accuracy peak are on the same c value. This c value is 2. So we finally choose c = 2 as an optimized parameter in our method.

**Table 3:** Prediction accuracy for different c

Location	c = 1	c = 2	c = 3	c = 4
Inner membrane	80.9%	**85.5%**	83.9%	82.4%
Outer membrane	51.2%	**51.2%**	36.5%	34.1%
Matrix	93.1%	94.5%	**95.9%**	93.8%

Over all	82.4%	**85.2%**	83.0%	81.1%

Another technique that had been rarely used previously in subcellular location prediction studies is the 9 kinds of physicochemical properties that we used in our method. As we described in Method section, only 1 kind of physicochemical property had been used in Chou's pseudo-amino acid composition. Here we show a comparison result to demonstrate the usefulness of the additional physicochemical properties. We exclude all physicochemical properties except "Hydrophilicity value" and "Consensus normalized hydrophobicity" and perform prediction with these 2 properties. The comparison result is shown in Table 4. We find that the decrease in accuracy is significant after we exclude 7 kinds of physicochemical properties, especially the accuracy at outer membrane location. We believe that this decrease in accuracy is the result of losing information about long distance interaction between residues along the sequence.

**Table 4:** Prediction accuracy for different number of physicochemical properties

Location	Using 9 properties	Using 2 properties
Inner membrane	85.5%	**86.3%**
Outer membrane	**51.2%**	29.3%
Matrix	**94.5%**	91.0%

Over All	**85.2%**	81.1%

### Software

The available data on submitochondria location in Swiss-Prot database increases rapidly, so we designed our software with an upgradeable architecture. The model we used in our software can be updated if a certain amount of new data is available. We will publish these updates on the web site of SubMito.

Another point we need to make it clear is that SubMito only predicts submitochondria location for a mitochondria protein. Users of SubMito should only submit known or predicted mitochondria protein to SubMito. If users only have an amino acid sequence, they should use MitoPred (which is the best mitochondria protein predictor in our opinion) to predict whether the sequence is a mitochondria protein first. If the user submits a predicted mitochondrial protein to SubMito, the program's rate of false positives will be higher, as some of the submitted proteins will be false positives generated by the mitochondrial prediction server.

## Conclusion

In this paper, we develop a computational system for predicting protein submitochondria location only from its primary sequence. Like subnuclear location prediction, submitocondria location predictor can predict the location of a protein with higher precision than subcellular location prediction. Online service and software SubMito has been developed for predicting protein submitochondria location. By reviewing similar work at the subcellular level, predicting submitochondria location is still a challenging problem.

## Methods

### Data set

The raw data set used in this work is extracted from Swiss-Prot[49] release 48.0. To construct a high quality working dataset, we use the following steps to process all sequences extracted from the database.

(1) The sequences which have a subcellular location annotation containing word "mitochondrion" are selected. The following steps are done on this subset of all sequences.

(2) The sequences which have a subcellular location annotation containing any of the words "Probable", "Potential", "Possible" or "By Similarity" are excluded, because their annotations are lack of confidence.

(3) The sequences containing ambiguous residues like "X", "B" and "Z" are excluded.

(4) The sequences which are fragment of other proteins are excluded.

(5) The sequences which localize at more than one submitochondria location are excluded.

(6) The left sequences are processed using the CD-HIT[50] program to remove the highly homologues sequences. The identity between any 2 sequences in the processed data set is less than 40%. The identity cut off is set to 40% in order to get a balance between the homologous bias and the size of the training set.

(7) The sequences localizing at inner membrane without membrane protein type annotation like "multi-pass membrane protein", "matrix side" or "peripheral membrane protein" are excluded.

(8) The submitochondria locations or the membrane protein type containing less than 15 sequences are dropped.

After strictly following the above steps, we finally obtain 317 sequences classified into 3 submitochondria locations. Table 5 shows the distribution of the data.

**Table 5:** The distribution of data set

Label	Compartment	Number of Sequence
1	Inner membrane	131
2	Outer membrane	41
3	Matrix	145

Total		317

The proteins localized at inner membrane are classified into 2 classes containing different membrane protein type. The "multi-pass membrane protein" has 101 sequences, and the "matrix side membrane protein" has 30 sequences.

### Feature vector

Proteins localized at different submitochondria locations have different N-terminal or C-terminal targeting signal peptides. Andrade, et al. [5] have pointed out that at the subcellular level, the average physicochemical properties of a protein molecular surface are adapted to the micro environment the protein localized at, and the average physicochemical properties of the molecular surface are correlated with the amino acid composition of the sequence. The investigation of Markov Chain[16] method and the work based on pseudo-amino acid composition[10] imply that the long distance interaction between residues is correlated with the subcellular location. We assume these are still correct at submitochondria level. So we attempt to construct a feature vector representing the targeting signal information, the average physicochemical properties of molecular surface and the long distance interactions between residues along the whole sequence.

The feature vector is made up by three parts. Before constructing the first two parts, the sequence is segmented into c same length segmentations.

The first part of the feature vector is the amino acid composition which is the occurrence frequencies of different residues. Assume the length of the ith segmentation is *L*_*i*_, and the numbers of different residues appear in the ith segmentation are *n*_1_, *n*_2_, ..., *n*_20_, the amino acid composition vector of the ith segmentation is defined in equation 4.

V→i1=1Li[n1,n2,⋯,n20]T     (4)
 MathType@MTEF@5@5@+=feaafiart1ev1aaatCvAUfKttLearuWrP9MDH5MBPbIqV92AaeXatLxBI9gBaebbnrfifHhDYfgasaacH8akY=wiFfYdH8Gipec8Eeeu0xXdbba9frFj0=OqFfea0dXdd9vqai=hGuQ8kuc9pgc9s8qqaq=dirpe0xb9q8qiLsFr0=vr0=vr0dc8meaabaqaciaacaGaaeqabaqabeGadaaakeaacuWGwbGvgaWcamaaBaaaleaacqWGPbqAcqaIXaqmaeqaaOGaeyypa0ZaaSaaaeaacqaIXaqmaeaacqWGmbatdaWgaaWcbaGaemyAaKgabeaaaaGccqGGBbWwcqWGUbGBdaWgaaWcbaGaeGymaedabeaakiabcYcaSiabd6gaUnaaBaaaleaacqaIYaGmaeqaaOGaeiilaWIaeS47IWKaeiilaWIaemOBa42aaSbaaSqaaiabikdaYiabicdaWaqabaGccqGGDbqxdaahaaWcbeqaaiabdsfaubaakiaaxMaacaWLjaWaaeWaaeaacqaI0aanaiaawIcacaGLPaaaaaa@49F8@

The amino acid composition may represent the average physicochemical properties of the molecular surface according to our assumptions, but the amino acid composition vector contains no sequence order information of the residues. We use the dipeptide composition which denotes the occurrence frequencies of two consecutive residues as the second part of the feature vector in order to add some sequence order information to the amino acid composition. Since we segment the sequence into c segmentations, this part of the feature vector may represent the sequence order information of different part of the sequence, especially the N-terminal and C-terminal targeting signal peptides. Assume that the numbers of different dipeptide appear in the ith segmentation are *n*_1_, *n*_2_, ..., *n*_400_, the dipeptide composition is defined in equation 5.

V→i2=1Li−1[n1,n2,⋯,n400]T     (5)
 MathType@MTEF@5@5@+=feaafiart1ev1aaatCvAUfKttLearuWrP9MDH5MBPbIqV92AaeXatLxBI9gBaebbnrfifHhDYfgasaacH8akY=wiFfYdH8Gipec8Eeeu0xXdbba9frFj0=OqFfea0dXdd9vqai=hGuQ8kuc9pgc9s8qqaq=dirpe0xb9q8qiLsFr0=vr0=vr0dc8meaabaqaciaacaGaaeqabaqabeGadaaakeaacuWGwbGvgaWcamaaBaaaleaacqWGPbqAcqaIYaGmaeqaaOGaeyypa0ZaaSaaaeaacqaIXaqmaeaacqWGmbatdaWgaaWcbaGaemyAaKgabeaakiabgkHiTiabigdaXaaacqGGBbWwcqWGUbGBdaWgaaWcbaGaeGymaedabeaakiabcYcaSiabd6gaUnaaBaaaleaacqaIYaGmaeqaaOGaeiilaWIaeS47IWKaeiilaWIaemOBa42aaSbaaSqaaiabisda0iabicdaWiabicdaWaqabaGccqGGDbqxdaahaaWcbeqaaiabdsfaubaakiaaxMaacaWLjaWaaeWaaeaacqaI1aqnaiaawIcacaGLPaaaaaa@4CCB@

After constructing the first two parts of the feature vector, the c segmentations are merged together to form a complete sequence again. The physicochemical properties of the residues are considered in the third part of the feature vector in order to involve some information about long distance interactions between residues. Chou used three kinds of physicochemical properties in his pseudo-amino acid composition[10,14,15], two kinds of properties in his amphiphilic pseudo-amino acid composition[25,38]. We choose 9 kinds of physicochemical properties which had been used in other researches[51,52] for our problem. We hope this will involve more information about the long distance interactions between residues along the sequence.

The first step to construct the third part of the feature vector is to replace the amino acid residues with the normalized amino acid indexes, which are numbers representing the physicochemical properties of the residue. The 9 physicochemical properties selected in our work are listed in Table 6. For the ith amino acid index extracted from AAIndex database[53], we use the normalization procedure described by equation 6 to 8 which has been used in Chou's hybridization space methods [54-56] to normalize physicochemical properties.

**Table 6:** The 9 physicochemical properties used in this work

Properties description	Reference
Hydrophilicity value	Hopp-Woods (1981)
Mean polarity	Radzicka-Wolfenden (1988)
Isoelectric point	Zimmerman et al .(1968)
Refractivity	McMeekin et al. (1964)
Average flexibility indices	Bhaskaran-Ponnuswamy (1988)
Average volume of buried residue	Chothia (1975)
Electron-ion interaction potential values	Cosic (1994)
Transfer free energy to surface	Bull-Breese (1974)
Consensus normalized hydrophobicity	Eisenberg (1984)

All the information in this table is derived from AAIndex database.

pnormal_i(k)=pi(k)−p¯iVar(pi)     (6)
 MathType@MTEF@5@5@+=feaafiart1ev1aaatCvAUfKttLearuWrP9MDH5MBPbIqV92AaeXatLxBI9gBaebbnrfifHhDYfgasaacH8akY=wiFfYdH8Gipec8Eeeu0xXdbba9frFj0=OqFfea0dXdd9vqai=hGuQ8kuc9pgc9s8qqaq=dirpe0xb9q8qiLsFr0=vr0=vr0dc8meaabaqaciaacaGaaeqabaqabeGadaaakeaacqWGWbaCdaqhaaWcbaGaemOBa4Maem4Ba8MaemOCaiNaemyBa0MaemyyaeMaemiBaWMaei4xa8LaemyAaKgabaGaeiikaGIaem4AaSMaeiykaKcaaOGaeyypa0ZaaSaaaeaacqWGWbaCdaqhaaWcbaGaemyAaKgabaGaeiikaGIaem4AaSMaeiykaKcaaOGaeyOeI0IafmiCaaNbaebadaWgaaWcbaGaemyAaKgabeaaaOqaamaakaaabaGaemOvayLaemyyaeMaemOCaiNaeiikaGIaemiCaa3aaSbaaSqaaiabdMgaPbqabaGccqGGPaqkaSqabaaaaOGaaCzcaiaaxMaadaqadaqaaiabiAda2aGaayjkaiaawMcaaaaa@53EC@

Where

p¯i=120∑k=120pi(k)     (7)
 MathType@MTEF@5@5@+=feaafiart1ev1aaatCvAUfKttLearuWrP9MDH5MBPbIqV92AaeXatLxBI9gBaebbnrfifHhDYfgasaacH8akY=wiFfYdH8Gipec8Eeeu0xXdbba9frFj0=OqFfea0dXdd9vqai=hGuQ8kuc9pgc9s8qqaq=dirpe0xb9q8qiLsFr0=vr0=vr0dc8meaabaqaciaacaGaaeqabaqabeGadaaakeaacuWGWbaCgaqeamaaBaaaleaacqWGPbqAaeqaaOGaeyypa0ZaaSaaaeaacqaIXaqmaeaacqaIYaGmcqaIWaamaaWaaabCaeaacqWGWbaCdaqhaaWcbaGaemyAaKgabaGaeiikaGIaem4AaSMaeiykaKcaaaqaaiabdUgaRjabg2da9iabigdaXaqaaiabikdaYiabicdaWaqdcqGHris5aOGaaCzcaiaaxMaadaqadaqaaiabiEda3aGaayjkaiaawMcaaaaa@44E5@

Var(pi)=120∑k=120(pi(k)−p¯i)2     (8)
 MathType@MTEF@5@5@+=feaafiart1ev1aaatCvAUfKttLearuWrP9MDH5MBPbIqV92AaeXatLxBI9gBaebbnrfifHhDYfgasaacH8akY=wiFfYdH8Gipec8Eeeu0xXdbba9frFj0=OqFfea0dXdd9vqai=hGuQ8kuc9pgc9s8qqaq=dirpe0xb9q8qiLsFr0=vr0=vr0dc8meaabaqaciaacaGaaeqabaqabeGadaaakeaacqWGwbGvcqWGHbqycqWGYbGCcqGGOaakcqWGWbaCdaWgaaWcbaGaemyAaKgabeaakiabcMcaPiabg2da9maalaaabaGaeGymaedabaGaeGOmaiJaeGimaadaamaaqahabaWaaeWaaeaacqWGWbaCdaqhaaWcbaGaemyAaKgabaGaeiikaGIaem4AaSMaeiykaKcaaOGaeyOeI0IafmiCaaNbaebadaWgaaWcbaGaemyAaKgabeaaaOGaayjkaiaawMcaamaaCaaaleqabaGaeGOmaidaaaqaaiabdUgaRjabg2da9iabigdaXaqaaiabikdaYiabicdaWaqdcqGHris5aOGaaCzcaiaaxMaadaqadaqaaiabiIda4aGaayjkaiaawMcaaaaa@511F@

For each property, the replacement produces a serial of numbers. Assume that for the ith property, the serial is p1(i)p2(i)⋯pL(i)
 MathType@MTEF@5@5@+=feaafiart1ev1aaatCvAUfKttLearuWrP9MDH5MBPbIqV92AaeXatLxBI9gBaebbnrfifHhDYfgasaacH8akY=wiFfYdH8Gipec8Eeeu0xXdbba9frFj0=OqFfea0dXdd9vqai=hGuQ8kuc9pgc9s8qqaq=dirpe0xb9q8qiLsFr0=vr0=vr0dc8meaabaqaciaacaGaaeqabaqabeGadaaakeaacqWGWbaCdaqhaaWcbaGaeGymaedabaGaeiikaGIaemyAaKMaeiykaKcaaOGaemiCaa3aa0baaSqaaiabikdaYaqaaiabcIcaOiabdMgaPjabcMcaPaaakiabl+UimjabdchaWnaaDaaaleaacqWGmbataeaacqGGOaakcqWGPbqAcqGGPaqkaaaaaa@3F9A@, where L is the length of the sequence and pk(i)
 MathType@MTEF@5@5@+=feaafiart1ev1aaatCvAUfKttLearuWrP9MDH5MBPbIqV92AaeXatLxBI9gBaebbnrfifHhDYfgasaacH8akY=wiFfYdH8Gipec8Eeeu0xXdbba9frFj0=OqFfea0dXdd9vqai=hGuQ8kuc9pgc9s8qqaq=dirpe0xb9q8qiLsFr0=vr0=vr0dc8meaabaqaciaacaGaaeqabaqabeGadaaakeaacqWGWbaCdaqhaaWcbaGaem4AaSgabaGaeiikaGIaemyAaKMaeiykaKcaaaaa@32AE@,1 ≤ *k *≤ *L *is the ith normalized amino acid index of the kth residue in the sequence. Then we calculate the value of auto correlation function *R*_*i*_(*τ*), 1 ≤ *τ *≤ *T *using equation 9, where T is a constant.

Ri(τ)=1L−τ∑k=1L−τpk(i)pk+τ(i)     (9)
 MathType@MTEF@5@5@+=feaafiart1ev1aaatCvAUfKttLearuWrP9MDH5MBPbIqV92AaeXatLxBI9gBaebbnrfifHhDYfgasaacH8akY=wiFfYdH8Gipec8Eeeu0xXdbba9frFj0=OqFfea0dXdd9vqai=hGuQ8kuc9pgc9s8qqaq=dirpe0xb9q8qiLsFr0=vr0=vr0dc8meaabaqaciaacaGaaeqabaqabeGadaaakeaacqWGsbGudaWgaaWcbaGaemyAaKgabeaakiabcIcaOGGaciab=r8a0jabcMcaPiabg2da9maalaaabaGaeGymaedabaGaemitaWKaeyOeI0Iae8hXdqhaamaaqahabaGaemiCaa3aa0baaSqaaiabdUgaRbqaaiabcIcaOiabdMgaPjabcMcaPaaakiabdchaWnaaDaaaleaacqWGRbWAcqGHRaWkcqWFepaDaeaacqGGOaakcqWGPbqAcqGGPaqkaaaabaGaem4AaSMaeyypa0JaeGymaedabaGaemitaWKaeyOeI0Iae8hXdqhaniabggHiLdGccaWLjaGaaCzcamaabmaabaGaeGyoaKdacaGLOaGaayzkaaaaaa@549D@

So for each property, we get the third part of the feature vector which may involve some information about the long distance interactions between residues along the sequence.

V→i3=[Ri(1),Ri(2),⋯,Ri(T)]T     (10)
 MathType@MTEF@5@5@+=feaafiart1ev1aaatCvAUfKttLearuWrP9MDH5MBPbIqV92AaeXatLxBI9gBaebbnrfifHhDYfgasaacH8akY=wiFfYdH8Gipec8Eeeu0xXdbba9frFj0=OqFfea0dXdd9vqai=hGuQ8kuc9pgc9s8qqaq=dirpe0xb9q8qiLsFr0=vr0=vr0dc8meaabaqaciaacaGaaeqabaqabeGadaaakeaacuWGwbGvgaWcamaaBaaaleaacqWGPbqAcqaIZaWmaeqaaOGaeyypa0ZaamWaaeaacqWGsbGudaWgaaWcbaGaemyAaKgabeaakiabcIcaOiabigdaXiabcMcaPiabcYcaSiabdkfasnaaBaaaleaacqWGPbqAaeqaaOGaeiikaGIaeGOmaiJaeiykaKIaeiilaWIaeS47IWKaeiilaWIaemOuai1aaSbaaSqaaiabdMgaPbqabaGccqGGOaakcqWGubavcqGGPaqkaiaawUfacaGLDbaadaahaaWcbeqaaiabdsfaubaakiaaxMaacaWLjaWaaeWaaeaacqaIXaqmcqaIWaamaiaawIcacaGLPaaaaaa@4E74@

Finally, three parts of the feature vector, the c amino acid composition vectors, c dipeptide composition vectors and 9 auto correlation vectors are combined to form a 420c+9T dimension feature vector as equation 11.

V→=[V→11,V→21,⋯,V→c1,V→12,V→22,⋯,V→c2,V→13,V→23,⋯,V→93]T     (11)
 MathType@MTEF@5@5@+=feaafiart1ev1aaatCvAUfKttLearuWrP9MDH5MBPbIqV92AaeXatLxBI9gBaebbnrfifHhDYfgasaacH8akY=wiFfYdH8Gipec8Eeeu0xXdbba9frFj0=OqFfea0dXdd9vqai=hGuQ8kuc9pgc9s8qqaq=dirpe0xb9q8qiLsFr0=vr0=vr0dc8meaabaqaciaacaGaaeqabaqabeGadaaakeaacuWGwbGvgaWcaiabg2da9maadmaabaGafmOvayLbaSaadaWgaaWcbaGaeGymaeJaeGymaedabeaakiabcYcaSiqbdAfawzaalaWaaSbaaSqaaiabikdaYiabigdaXaqabaGccqGGSaalcqWIVlctcqGGSaalcuWGwbGvgaWcamaaBaaaleaacqWGJbWycqaIXaqmaeqaaOGaeiilaWIafmOvayLbaSaadaWgaaWcbaGaeGymaeJaeGOmaidabeaakiabcYcaSiqbdAfawzaalaWaaSbaaSqaaiabikdaYiabikdaYaqabaGccqGGSaalcqWIVlctcqGGSaalcuWGwbGvgaWcamaaBaaaleaacqWGJbWycqaIYaGmaeqaaOGaeiilaWIafmOvayLbaSaadaWgaaWcbaGaeGymaeJaeG4mamdabeaakiabcYcaSiqbdAfawzaalaWaaSbaaSqaaiabikdaYiabiodaZaqabaGccqGGSaalcqWIVlctcqGGSaalcuWGwbGvgaWcamaaBaaaleaacqaI5aqocqaIZaWmaeqaaaGccaGLBbGaayzxaaWaaWbaaSqabeaacqWGubavaaGccaWLjaGaaCzcamaabmaabaGaeGymaeJaeGymaedacaGLOaGaayzkaaaaaa@6595@

After several testing, we found that c = 2 and T = 20 are the best parameters for the prediction.

### Prediction algorithm

SVM is machine learning algorithm based on Statistical Learning Theory which was introduced by Vapnik. It searches for an optimal separating hyper plane which maximizes the margin in feature space. SVM was originally introduced to solve binary classification problem. A one-versus-one framework was adopted in this work to deal with the multi-class classification problem. Altogether 4 classifiers were designed using SVM. For every two locations listed in Table 7, we construct a classifier, and for two different membrane protein types at inner membrane, the 4th classifier is constructed.

**Table 7:** The classifiers parameters and accuracy

Classifier	C	γ	Leave-one-out accuracy
inmem_otmem	100	0.001	90.7%
inmem_matrx	100	0.005	90.9%
matrx_otmem	100	0.001	91.4%
mlps_mtrx	100	0.007	92.4%

The parameter C and γ are manually searched to get as high accuracy as possible. The "inmem" means inner membrane, "otmem" means outer membrane, "matrx" means matrix, "mlps" means multi-pass membrane and "mtrx" means the matrix side.

Since the RBF kernel is the most flexible and the most widely used kernel function, a RBF kernel function is used in our classifier. The RBF kernel function is described as the following:

K(x→i,x→j)=exp⁡(−γ‖x→i−x→j‖2)     (12)
 MathType@MTEF@5@5@+=feaafiart1ev1aaatCvAUfKttLearuWrP9MDH5MBPbIqV92AaeXatLxBI9gBaebbnrfifHhDYfgasaacH8akY=wiFfYdH8Gipec8Eeeu0xXdbba9frFj0=OqFfea0dXdd9vqai=hGuQ8kuc9pgc9s8qqaq=dirpe0xb9q8qiLsFr0=vr0=vr0dc8meaabaqaciaacaGaaeqabaqabeGadaaakeaacqWGlbWsdaqadaqaaiqbdIha4zaalaWaaSbaaSqaaiabdMgaPbqabaGccqGGSaalcuWG4baEgaWcamaaBaaaleaacqWGQbGAaeqaaaGccaGLOaGaayzkaaGaeyypa0JagiyzauMaeiiEaGNaeiiCaa3aaeWaaeaacqGHsisliiGacqWFZoWzdaqbdaqaaiqbdIha4zaalaWaaSbaaSqaaiabdMgaPbqabaGccqGHsislcuWG4baEgaWcamaaBaaaleaacqWGQbGAaeqaaaGccaGLjWUaayPcSdWaaWbaaSqabeaacqaIYaGmaaaakiaawIcacaGLPaaacaWLjaGaaCzcamaabmaabaGaeGymaeJaeGOmaidacaGLOaGaayzkaaaaaa@4FF2@

where x→
 MathType@MTEF@5@5@+=feaafiart1ev1aaatCvAUfKttLearuWrP9MDH5MBPbIqV92AaeXatLxBI9gBaebbnrfifHhDYfgasaacH8akY=wiFfYdH8Gipec8Eeeu0xXdbba9frFj0=OqFfea0dXdd9vqai=hGuQ8kuc9pgc9s8qqaq=dirpe0xb9q8qiLsFr0=vr0=vr0dc8meaabaqaciaacaGaaeqabaqabeGadaaakeaacuWG4baEgaWcaaaa@2E37@_*i *_and x→
 MathType@MTEF@5@5@+=feaafiart1ev1aaatCvAUfKttLearuWrP9MDH5MBPbIqV92AaeXatLxBI9gBaebbnrfifHhDYfgasaacH8akY=wiFfYdH8Gipec8Eeeu0xXdbba9frFj0=OqFfea0dXdd9vqai=hGuQ8kuc9pgc9s8qqaq=dirpe0xb9q8qiLsFr0=vr0=vr0dc8meaabaqaciaacaGaaeqabaqabeGadaaakeaacuWG4baEgaWcaaaa@2E37@_*j *_are feature vectors, and γ is a parameter.

We use a grid search approach assisted by manually trial to find a good parameter combination for C and γ for each classifier, where C is the cost parameter of SVM and γ is the parameter in RBF kernel function. The results of parameter optimization and leave-one-out cross validation accuracy for the four classifiers are shown in Table 3.

While predicting submitochondria location for a test sample, the first 3 classifiers take a vote on the test sample. The test sample gets a score for each of the 3 submitochondria locations. And it will predict the location as being that with the highest score. If the three locations have the same score, the predictor reports "unknown" as a result. If the test sample is predicted to localize at inner membrane then the forth classifier predicts the membrane protein type for the test sample.

## Availability and requirements

Project name: SubMito.

Project home page: .

Operating system: online service is web based; local version of the software is platform independent.

Programming language: Java and PHP.

Other requirements: online service needs a web browser supporting JavaScript. Local version of the software needs Java Runtime Environment version higher than 1.5.0.

License: free.

For non-academics use, please contact daulyd@tsinghua.edu.cn.

## Authors' contributions

PD extracts the data from Swiss-Prot database, implements the algorithm, carries out the analyses and writes the manuscript. YL guides the design of the study, analyses the data and result and writes the manuscript. All authors read and approve the final manuscript.
